# Adolescent E-cigarette use, social media exposure and socioeconomic inequality – results from a german school-based survey

**DOI:** 10.1016/j.pmedr.2026.103398

**Published:** 2026-01-31

**Authors:** Maike Trümpelmann, Mareike Lüthgen, Daniel Drömann, Folke Brinkmann, Loana Penner, Annika Burgard, Paul Axt, Henrike A. Faesser, Tobias Jagomast, Ulf Bachmann, Anna-Christina Sondersorg, Sabine Bohnet, Klaas F. Franzen

**Affiliations:** a Medical Clinic III (Pulmonology), Campus Lübeck, University Hospital Schleswig-Holstein, 23562 Lübeck, Germany; b Airway Research Center North, Member of the German Center for Lung Research (DZL), 22927 Großhansdorf, Germany; c Grund- und Gemeinschaftsschule Sandesneben mit gymnasialer Oberstufe, Sandesneben, Germany; d Carl-Jacob-Burckhardt Gymnasium, Lübeck, Germany; e Department of Pediatrics, University Hospital Schleswig-Holstein, Campus Lübeck, Lübeck, Germany

**Keywords:** Adolescents, E-cigarettes, Social media, Health literacy, Prevention, Participatory research

## Abstract

**Objectives:**

Adolescent e-cigarette use has increased worldwide, reflecting digital exposure and shifting norms. This study aimed to identify socioeconomic and digital determinants of e-cigarette use among adolescents and to inform prevention strategies.

**Methods:**

Data were collected from April to July 2024 among 829 students aged 11 to 19 years in three schools in northern Germany. An age-adapted questionnaire co-developed with students assessed sociodemographic characteristics, parental and media influences, risk perception, and nicotine use. Descriptive statistics and logistic regression models were applied.

**Results:**

Of all participants, 37.2% had tried e-cigarettes and 6.7% reported daily use. *E*-cigarette use increased with age (*p* < .01) but did not differ by gender. Exposure to e-cigarette content on TikTok (OR = 3.32, p < .01) and Instagram (OR = 3.00, p < .01) showed associations with e-cigarette use. Higher pocket money and parental nicotine consumption were associated with increased odds of e-cigarette use (OR = 3.00, *p* < .01; OR = 4.05, p < .01). Only 41% reported receiving school-based education on e-cigarette use**.**

**Conclusions:**

Adolescent e-cigarette use is shaped by digital exposure, socioeconomic resources, and parental behavior. Prevention requires regulation of advertising, integration into school curricula, and family-based communication.

## Highlights

• Nearly half of adolescents tried e-cigarettes; age, not gender, predicts use.
• About half of older teens saw nicotine content on TikTok/Instagram.
• Parental nicotine use and higher pocket money raise risk; reading and education protect.
• Only 41% report school discussion of vaping, revealing a major prevention gap.
• Participatory surveys are feasible; policy should curb algorithmic vaping promotion.

## 1 Introduction

Tobacco use remains one of the leading preventable causes of morbidity and mortality worldwide (Perez-Warnisher et al., 2018). Although adolescent smoking has declined in many high-income countries (Mauz et al., 2018), the use of e-cigarettes has risen and become the most common form of nicotine consumption among adolescents (Hanewinkel and Hansen, 2023).

The long-term health consequences of e-cigarette use remain uncertain, yet short-term evidence indicates respiratory risks (McConnell et al., 2017). Nicotine exposure during adolescence can impair brain development and increase dependency (Prochaska et al., 2022). These biological vulnerabilities are intertwined with social factors shaped by socioeconomic inequality, digital marketing, and family dynamics.

Across Europe, the link between socioeconomic status (SES) and adolescent e-cigarette use is complex. While lower parental education predicts higher tobacco use, adolescents from higher-income families appear more likely to try e-cigarettes, possibly due to greater purchasing power and online exposure (Azagba et al., 2023a; Kinnunen et al., 2021; Tarasenko et al., 2022).

The family environment remains decisive: parental smoking or e-cigarette use increases adolescent e-cigarette use through behavioral modelling and permissive norms (Sun et al., 2022; Trucco et al., 2021). Social media further amplifies risk. Although most forms of tobacco and e-cigarette advertising are restricted in Germany and the EU, Platforms such as TikTok or Instagram expose adolescents to advertising and user-generated e-cigarette use content, increasing initiation and lowering perceived harm (Adekeye et al., 2025; Cho et al., 2019; Vassey et al., 2022; Aly et al., 2022; Zheng et al., 2021).

The aim of this study is to identify behavioral and socioeconomic risk factors for e-cigarette use among adolescents in Germany and to develop evidence-based, school-centered prevention strategies.

## 2 Methods

### 2.1 Study design and setting

This cross-sectional study was conducted as part of the School*Vape*Science project, an educational and research initiative combining scientific literacy and nicotine prevention among adolescents. The project was implemented across three secondary schools in Lübeck, Germany and its surrounding districts between April and July 2024. The three participating schools represented urban, suburban, and rural contexts and included three types of German secondary education, with one academic-track school and two comprehensive schools, to capture socioeconomic heterogeneity.

The program followed a mixed-format design comprising (i) a six-week elective course introducing scientific methods, data collection, and health literacy and (ii) 90-min preventive teaching sessions delivered to all students secondary schools. Within this programm, students actively participated in the co-development and evaluation of a digital questionnaire designed to assess e-cigarette and cigarette use behaviors, socioeconomic determinants, and health perceptions. The students who helped with co-development of the survey questionnaire were not allowed to be participants in the actual survey study. Participation in the elective course varied by school and depended on local organisational structures: at two schools, the course was mandatory, while at one school participation was voluntary. No academic credit, grades, certificates, or other incentives were provided.

### 2.2 Participatory questionnaire development and validation

A central element of this study was the participatory development of the survey instrument, which was designed to ensure age appropriateness, content validity, and comprehension. The questionnaire was constructed iteratively using the LimeSurvey software, following a multi-step validation process that involved both expert and student inputs.

Initially, the research team, comprising medical professionals developed an item pool based on established European surveys and validated instruments for adolescent nicotine use (e.g., GYTS, KiGGS, and ESPAD). The draft items were refined for clarity and relevance during two internal review rounds.

In the next stage, the instrument was piloted with participating students in the elective course. These students completed the questionnaire and provided structured written and verbal feedback regarding comprehensibility, sensitivity of topics, and adequacy of answer options. Based on their feedback, the question wording and item order were revised to enhance clarity, age appropriateness, and engagement. Students were also invited to suggest additional items reflecting their lived experiences with e-cigarette use and social media exposure, aligning with a participatory research approach that integrated adolescent perspectives into health surveillance design.

Two versions of the instrument were created to accommodate the developmental and cognitive differences.

• a short form for grades 5–7 (students in this grades are usually between 10 and 19 years old), including 28 items.
• an extended form for grades 8–12 (students in this grades are usually between 13 and 19 years old), including 40 items.

Both versions covered the following key domains: sociodemographic and socioeconomic characteristics, household and parental factors, nicotine consumption, media use and advertising exposure, perceived risk and harm, and self-reported physical and mental health indicators. Media exposure was assessed as self-reported perceived exposure to nicotine-related content across multiple channels (e.g. television, billboards, YouTube, TikTok, Instagram, print and audio media). The instrument intentionally did not distinguish between formally labelled advertising, influencer content, or user-generated material, as these boundaries are often blurred and difficult for adolescents to identify. The measure therefore captures perceived promotional exposure rather than legally defined advertising. TikTok and Instagram were included as predefined platforms based on prior consultation with participating student groups regarding their actual media use. Platform use was assessed separately from content exposure.

Internal validation focuses on face and content validity through iterative feedback cycles. Reliability testing was performed on the selected multi-item constructs (Cronbach's α > 0.75 for attitudinal and media exposure scales). This participatory validation process was central to ensuring that the questionnaire reflected students' linguistic, cultural, and experiential realities, in line with recommendations for inclusive and age-sensitive public health research (Brandt et al., 2019; Gaiha et al., 2021).

### 2.3 Study population

All the students enrolled in grades 5–12 at the three participating schools were invited to complete the survey. In Germany, students usually start secondary school with 10–11 years in grade 5. Participation was voluntary and anonymous. In total, 977 questionnaires were submitted. Of these, 148 were excluded because of incomplete or inconsistent responses, yielding a final analytical sample of 829 students (response rate: 84.9%).

The sample was stratified into two cohorts: younger (grades 5–7, age usually between 10 and 13; *n* = 209) and older (grades 8–12, age usually between 13 and 19; *n* = 620). This stratification reflected differences in cognitive development and exposure risk following previous European public health surveys on adolescent substance use (Kinnunen et al., 2021; Tarasenko et al., 2022).

### 2.4 Measures

Data were collected using digital tablets and smartphones via a QR-coded survey access. Each student completed the questionnaire individually during the supervised classroom sessions to minimize peer influence.

Additionally, two optional physiological assessments were conducted for educational purposes within the School*Vape*Science Framework:

• Arterial stiffness and central hemodynamics were measured using the Mobil-O-Graph® device, providing real-time feedback through a color-coded “traffic light” system (green–yellow–red) to enhance health awareness.
• Small airway function was evaluated using a Tremoflo® device to assess respiratory health in relation to nicotine exposure.

These measurements were performed under professional supervision, fostering both hands-on scientific experience and reflection on individual health indicators.

### 2.5 Ethical considerations

The study was conducted in accordance with the Declaration of Helsinki and was approved by the Ethics Committee of the University of Lübeck (AZ 2024–267). Participation required informed consent from both students and their legal guardians. All responses were anonymized.

### 2.6 Statistical analysis

Data cleaning included removal of incomplete cases, consistency checks, and recording of categorical variables. Descriptive statistics were calculated to summarize demographic, behavioral, and socioeconomic characteristics.

Associations between e-cigarette use behavior and key predictors (age, sex, parental smoking, pocket money, education level, and social media exposure) were analyzed using logistic regression models adjusted for age, gender and school. Ordinal logistic regression was applied to estimate the trends in e-cigarette use frequency, and binary logistic regression was used to differentiate between students using e-cigarettes (≥1 use per month) and students who don't use e-cigarettes. Model fit was assessed using the Nagelkerke pseudo-R^2^ and Hosmer–Lemeshow tests. Statistical significance was set at *p* < .05. Effect estimates were reported as adjusted odds ratios. Visualizations were generated to facilitate the interpretation and support the policy relevance of the findings. Data analyses were conducted using the IBM SPSS Statistics (version 29).

## 3 Results

### 3.1 Study cohort

**Fig. 1:**
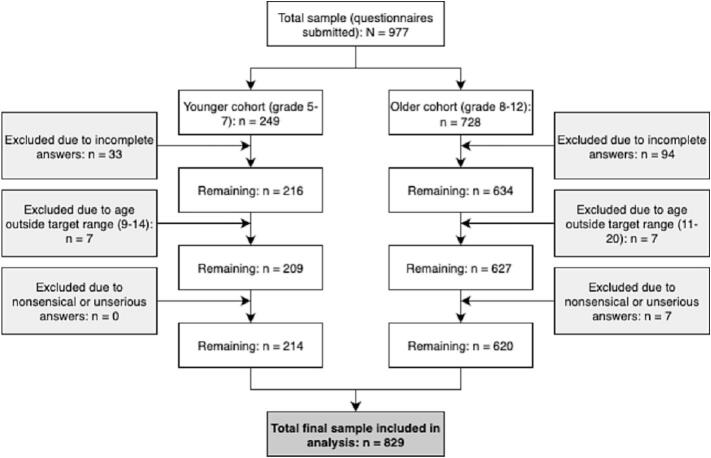
Flowchart of participant selection and exclusion in a school-based survey of adolescents in Northern Germany (2024).

In total, 977 questionnaires were submitted. After excluding incomplete, inconsistent, or out-of-range responses, 829 participants were included in the final analysis (response rate: 84.9%), (Fig. 1).

The study sample was divided into two educational cohorts.

• Younger cohort: Grades 5–7 (age: 11 to 14; *n* = 209; 103 male, 102 female, 4 diverse).
• Older cohort: Grades 8–12 (age: 13 to 19; *n* = 620; 303 males, 311 females, 6 diverse).

No significant differences in e-cigarette use were found between male and female students (*p* > .05). In contrast, age was strongly associated with e-cigarette consumption; with each additional year of age, the probability of e-cigarette use increased significantly (OR = 1.31, *p* < .01).

### 3.2 Prevalence of *E*-cigarette use by age and school cohort

**Fig. 2:**
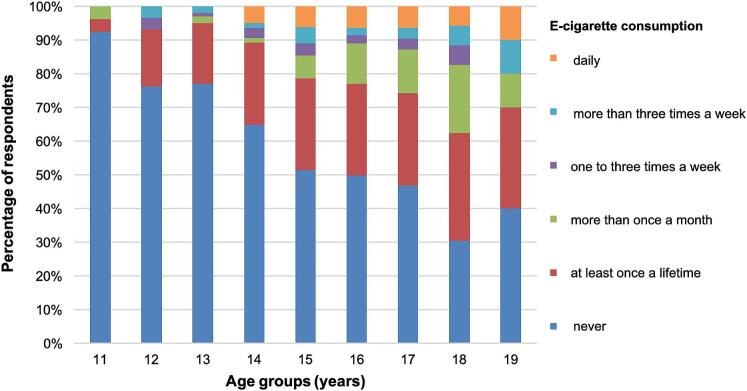
Frequency of e-cigarette use by age among adolescents in Northern Germany (2024).

In the younger cohort 24.4% reported ever using e-cigarettes. In the older cohort, 49.0% had used e-cigarettes at least once. A clear age-related gradient was observed (Table 1 and Fig. 2). Daily e-cigarette use remained comparatively uncommon (6.7% over all age groups), whereas experimentation was more common (37.2% in all age groups).

**Table 1:** Frequency of E-Cigarette Consumption by Age Group in Adolescents in Northern Germany (2024).

**Age**	**E-Cigarette consumption**						
	never	at least once a lifetime	more than once a month	one to three times a week	more than three times a week	daily	Total
11	24	1	1	0	0	0	26
12	45	10	0	2	2	0	59
13	77	18	2	1	2	0	100
14	90	34	2	4	2	7	139
15	98	52	13	7	9	12	191
16	86	47	21	4	4	11	173
17	29	17	8	2	2	4	62
18	21	22	14	4	4	4	69
19	4	3	1	0	1	1	10
Total	474	204	62	24	26	39	829

### 3.3 Exposure to advertising and social media

Social media use was highly prevalent in both cohorts. Among younger students, 59.3% used TikTok and 42.3% used Instagram, compared with 80.7% and 87.2% among older students, respectively. In the older cohort, 49.7% reported exposure to nicotine-related content on TikTok, 41.1% on Instagram, and 46.6% on YouTube. Exposure via traditional media was reported for television (54.0%) and billboards (55.0%), while newspapers (13.9%), podcasts (5.3%), and radio (4.7%) were less frequently mentioned.

**Fig. 3:**
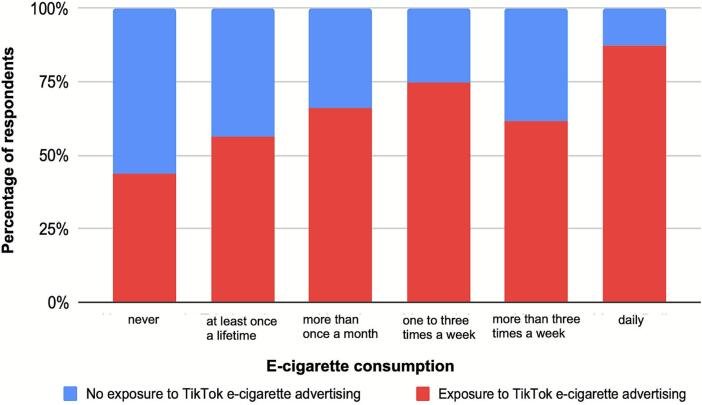
Association between exposure to e-cigarette–related content on TikTok and e-cigarette use among adolescents in Northern Germany (2024).

Students who reported exposure to the TikTok e-cigarette use content were significantly more likely to use e-cigarettes (OR = 3.32, *p* < .01) (Fig. 3).

Similarly, exposure to Instagram advertisements showed a strong positive association (OR = 3.00, p < .01), whereas television advertising was inversely correlated (OR = 0.54, *p* = .02). Podcast (OR = 3.00, p = .02) and newspaper (OR = 1.92, *p* = .04) exposure was also positively associated, although less frequently.

### 3.4 Economic and social determinants

The distribution of monthly pocket money differed markedly between age groups. Among students in grades 5–7 (age: 11 to 14), the majority received between €10 and €50 per month (68.8%), while only 6.7% received more than €50. In grades 8–12 (age: 13 to 19), the proportion receiving €50–100 or more was considerably higher (26.4%), and nearly half (43.7%) received €20–50. In both cohorts, 16.7% reported not receiving any pocket money. Students reporting €50–100 or more per month had a significantly higher probability of e-cigarette use compared to those with lower or no pocket money (OR = 3.00, *p* < .01).

Regarding reading behavior, 43.0% of younger students and 57.6% of older students reported reading no books per month. Reading one to two or three to four books per month was associated with reduced odds of e-cigarette use (OR = 0.50, *p* = .03; OR = 0.48, *p* = .02), suggesting that reading and academic engagement may act as protective factors.

In households with older students, higher parental education showed a protective, non-significant trend against adolescent e-cigarette use, particularly for university and doctoral degrees (*p* = .07 and *p* = .09, respectively).

### 3.5 Parental nicotine use

Parental nicotine consumption was reported by 47.4% of younger participants and 45.1% of older participants. Among them, 23.9% (younger) and 26.8% (older) reported smoking or e-cigarette use in their presence. Adolescents exposed to parental nicotine use in their presence was significantly associated with higher odds of e-cigarette use (OR = 4.05, *p* < .01).

### 3.6 Risk perception and school-based education

The perceived health risk of e-cigarettes varied notably between cohorts. Among younger students, 22.6% considered e-cigarettes to be “healthier” than tobacco cigarettes, while 37.7% believed they were not. In the older cohort, 25.4% believed e-cigarette use to be less harmful, 49.4% to be equally harmful, and 25.1% to be more harmful. A higher perception of harm was significantly associated with a lower probability of e-cigarette use (OR = 0.66, *p* < .05). Despite this, only 41.1% of the students reported that e-cigarettes had been discussed in school, indicating a gap in formal prevention education.

## 4 Discussion

This school-based cross-sectional study of 829 adolescents in northern Germany identified several key determinants of e-cigarette use. Age was the strongest predictor, while no gender differences were observed. Exposure to social media advertising, particularly TikTok and Instagram, was robustly associated with higher odds of e-cigarette use. Higher monthly pocket money and parental nicotine consumption further increased the risk, whereas regular reading habits were inversely associated with e-cigarette use. Higher household education showed a protective trend. Moreover, adolescents who perceived e-cigarette use as harmful were less likely to use e-cigarettes, whereas school-based prevention coverage remained low. Together, these results depict a multifactorial risk environment in which digital media exposure, socioeconomic context, and family modelling interact to shape adolescent nicotine behavior.

The observed associations between social media use and e-cigarette use reflect growing international evidence that digital advertising environments function as causal drivers of nicotine use in adolescents (Cho et al., 2019; Lee, 2021; Vassey et al., 2022). This finding supports prior mediation models in which media exposure lead to reduced harm perception and increased use (Hung et al., 2022; Li et al., 2024; Stanton et al., 2022). Parental nicotine use has emerged as a strong determinant, which is consistent with robust evidence. Adolescents whose parents smoke or use e-cigarettes are twice as likely to use e-cigarettes themselves (Sun et al., 2022; Yan et al., 2024). This causal influence operates through behavioral modelling, permissive attitudes, and reduced parental monitoring (Mantey et al., 2022; Sabbagh et al., 2025; Trucco et al., 2021). Our data strengthen this interpretation: *E*-cigarette use was significantly more frequent among adolescents exposed to parental nicotine consumption at home. Therefore, preventive efforts should not focus exclusively on individual behavior but also target family based interventions, promoting clear household rules and open communication regarding e-cigarette use risks.

The role of SES in e-cigarette use behavior remains complex and context-dependent. Several European studies report inconsistent associations between SES and e-cigarette use, often mediated by access to disposable income, academic achievement, or advertising exposure (Kinnunen et al., 2021; Tarasenko et al., 2022). While lower parental education is a well-established risk factor for tobacco smoking (Kuntz et al., 2018), e-cigarette use may follow a reversed or ambivalent gradient, with adolescents from higher-income households experimenting more because of greater purchasing power and digital connectivity (Azagba et al., 2023b; Chen et al., 2024). Our findings support this nuanced perspective: higher pocket money was significantly associated with e-cigarette use, whereas parental education was not significantly associated but showed a weak protective trend. This suggests that financial resources act as enabling factors rather than as direct causal determinants. Regulatory strategies should, therefore, include strict enforcement of age verification and sales restrictions, particularly in online retail, which adolescents frequently access via social media channels (Gaiha et al., 2025).

Reading frequency emerged as an inverse correlation of e-cigarette use, consistent with the concept of health literacy as a protective resource. European studies have shown that adolescents capable of critically appraising and applying health information, rather than merely retrieving it, are less likely to engage in substance use (Brandt et al., 2019). This protective link likely reflects broader cognitive and social engagement, academic motivation and resistance to manipulative advertising. The School*Vape*Science initiative underscores the potential of educational interventions that strengthen critical thinking and media literacy to buffer the influence of social media marketing.

Evidence on school-based e-cigarette use prevention remains mixed: most programs improve knowledge and attitudes but do not always sustain behavioral outcomes (Gardner et al., 2024; Mylocopos et al., 2024). Interventions combining interactive learning, peer-led elements, and refusal skills training, as described in the existing literature (e.g. YES-CAN!, MediaSense), show the most promise (Asdigian et al., 2023; Michaud et al., 2025). While this study employed a participatory design, it did not systematically assess students' views on specific prevention strategies. Informal discussions suggested that social media content and peer norms were perceived as influential; however, specific educational formats were not evaluated.

Importantly, only 41% of our participants reported that e-cigarettes had been discussed in school, confirming a prevention gap in the formal curricula. Scaling up participatory, media-literacy-driven programs could thus strengthen both health literacy and empowerment, key priorities within European Union youth health promotion strategies. These implications for European public health policy are particularly relevant given that the convergence of digital marketing exposure, socioeconomic resources, and family modelling suggests that adolescent e-cigarette use reflects broader shifts in the social determinants of health within the digital age.

Therefore, effective prevention must operate on multiple levels:

1. Regulatory action on digital advertising and algorithms: Enforcement of age gating and transparent moderation of e-cigarette-related content on TikTok and Instagram under the European Digital Services Act is essential to reduce adolescent exposure.
2. Integration of media literacy and critical thinking in school curricula: Evidence indicates that enhancing media-literacy competencies can mitigate susceptibility to e-cigarette use (Gardner et al., 2024; Michaud et al., 2025).
3. Family oriented prevention: Programs that engage parents to establish clear norms, model healthy behavior, and discuss e-cigarette use risks to reduce initiation odds (Mantey et al., 2022; Trucco et al., 2021).
4. Addressing financial accessibility: Stricter control of online sales and education on responsible spending among adolescents can limit purchasing opportunities.

Such integrated policies align with the WHO Framework Convention on Tobacco Control (FCTC, Article 12) and the European Union Beating Cancer Plan, both of which emphasize adolescent-targeted education and advertising restrictions.

The strengths of this study include its large and socioeconomically diverse school sample; its integration of behavioral, socioeconomic, and digital determinants; and its participatory questionnaire development with iterative student feedback, enhancing content validity and acceptance. The limitations include the regional sampling frame, which may limit generalizability. As data collection was restricted to the greater Lübeck area, the generalisability of the results to international contexts and to large metropolitan regions should be interpreted with caution. A further limitation is that exposure was measured as perceived exposure to nicotine-related content and did not differentiate between advertising, influencer, or user-generated content, limiting conclusions about specific regulatory categories. In addition, the study focused on publicly accessible platforms; private messaging and peer-to-peer channels were not assessed. Another limitation is the cross-sectional design which cannot determine the temporal sequence of media exposure, perception change, and initiation, although longitudinal evidence from prior studies supports this causal direction (Vassey et al., 2022; Zheng et al., 2021). Future research should incorporate longitudinal follow-up and mixed-method approaches to explore nuanced mechanisms and evaluate the intervention effects over time.

## 5 Conclusion

*E*-cigarette use among adolescents is shaped by intersecting digital, socioeconomic, and familial determinants, with social media exposure emerging as the strongest risk factor. Protective influences, such as reading habits and household education, highlight the importance of health literacy. Effective prevention requires a multilevel approach: stricter digital advertising regulation, school-based media-literacy programs, and parental engagement.

## CRediT authorship contribution statement

**Maike Trümpelmann:** Writing – review & editing, Writing – original draft, Visualization, Resources, Investigation, Formal analysis, Data curation, Conceptualization. **Mareike Lüthgen:** Writing – review & editing, Writing – original draft, Visualization, Supervision, Project administration, Methodology, Investigation, Formal analysis, Data curation, Conceptualization. **Daniel Drömann:** Supervision, Project administration, Funding acquisition, Conceptualization. **Folke Brinkmann:** Writing – review & editing, Validation, Supervision, Project administration, Methodology, Investigation, Funding acquisition, Formal analysis, Conceptualization. **Loana Penner:** Writing – review & editing, Supervision, Project administration, Methodology, Investigation, Formal analysis, Data curation, Conceptualization. **Klaas F. Franzen:** Writing – review & editing, Supervision, Project administration, Methodology, Investigation, Funding acquisition, Formal analysis, Data curation, Conceptualization. **Annika Burgard:** Investigation, Data acquisition. **Paul Axt:** Investigation, Data acquisition, Formal analysis. **Henrike A. Faesser:** Investigation, Data acquisition. **Tobias Jagomast:** Investigation, Data acquisition. **Ulf Bachmann:** Investigation, Data acquisition, Methodology, Conceptualization, Supervision. **Anna-Christina Sondersorg:** Investigation, Data acquisition, Conceptualization. **Sabine Bohnet:** Investigation, Data acquisition, Supervision.

## Funding

The authors received financial support for the project from the Wessel- and Friedrich Bluhme and Else Jebsen-Foundation.

## Declaration of competing interest

The authors declare that they have no known competing financial interests or personal relationships that could have appeared to influence the work reported in this paper.

## Data Availability

Data will be made available on request.
